# Electrodeposition of Sn-Ru Alloys by Using Direct, Pulsed, and Pulsed Reverse Current for Decorative Applications

**DOI:** 10.3390/ma17215326

**Published:** 2024-10-31

**Authors:** Margherita Verrucchi, Giulio Mazzoli, Andrea Comparini, Roberta Emanuele, Marco Bonechi, Ivan Del Pace, Walter Giurlani, Claudio Fontanesi, Remigiusz Kowalik, Massimo Innocenti

**Affiliations:** 1 Department of Chemistry “Ugo Schiff”, University of Florence, Via della Lastruccia 3, 50019 Sesto Fiorentino, FI, Italy; 2 Valmet Plating Srl, Via Erbosa 5, 50041 Calenzano, FI, Italy; 3 National Interuniversity Consortium of Materials Science and Technology (INSTM), Via G. Giusti 9, 50121 Firenze, FI, Italy; 4 Department of Engineering “Enzo Ferrari” (DIEF), University of Modena and Reggio Emilia, Via Vivarelli 10, 41125 Modena, Italy; 5 Faculty of Non-Ferrous Metals, AGH University of Krakow, al. Mickiewicza 30, 30-059 Krakow, Poland; 6 National Research Council-Organometallic Compounds Chemistry Institute (CNR-ICCOM), Via Madonna del Piano 10, 50019 Sesto Fiorentino, FI, Italy; 7 Center for Colloid and Surface Science (CSGI), Via della Lastruccia 3, 50019 Sesto Fiorentino, FI, Italy

**Keywords:** electrodeposition, pulsed current, Sn-Ru alloys, anti-corrosive, decorative applications

## Abstract

Pulsed current has proven to be a promising alternative to direct current in electrochemical deposition, offering numerous advantages regarding deposit quality and properties. Concerning the electrodeposition of metal alloys, the role of pulsed current techniques may vary depending on the specific metals involved. We studied an innovative tin–ruthenium electroplating bath used as an anti-corrosive layer for decorative applications. The bath represents a more environmentally and economically viable alternative to nickel and palladium formulations. The samples obtained using both direct and pulsed currents were analyzed using various techniques to observe any differences in thickness, color, composition, and morphology of the deposits depending on the pulsed current waveform used for deposition.

## 1. Introduction

In recent years, the electroplating industry has been prompted to investigate environmentally sustainable and cost-effective solutions due to the volatility in the pricing of precious metals and the escalating environmental concern over using hazardous compounds [1,2,3,4]. The industry of fashion accessories, for example, has identified new nickel-free electroplating sequences to overcome the increasingly stringent legislation regulating the use of this metal, especially in the production of wearables, because of its allergenic properties [5,6]. The potential substitution of nickel and the selection of an alternative deposition material is intricately tied to its specific function within the galvanic sequence and the characteristics of the desired metal or metal alloy replacement. Currently, the prevalent nickel-free sequence applied to substrates such as brass, bronze, or zamak involves, after the acid copper layer, the deposition of a thick layer of bronze and then a thinner layer of Pd to block intermetallic diffusion [6]. Nickel has historically been and continues to be widely employed for its corrosion-resistant attributes, particularly in the form of nickel–phosphorus (NiP) deposits [7,8,9]. Anti-corrosive layers are typically applied immediately preceding the precious metal deposition to inhibit the progression of corrosive processes on the surface. Indeed, despite the theoretical protective role of noble metals against the corrosion of underlying layers, the precious deposits often exhibit thin (because of the tendency of manufacturers to use as little noble metal as possible) or rough characteristics, leading to microcracks that expose the underlying metals to the atmosphere [6,10]. Additionally, the diffusion of metals from the substrate can alter the nobility level of a metal or alloy over time, thereby modifying its inherent properties [6,10]. Zinc–cobalt (Zn-Co) alloys find application as protective layers owing to their anti-corrosive properties, extending beyond decorative electroplating in various sectors [11]. Nevertheless, their environmental sustainability is compromised by the ecological implications associated with cobalt usage. Pure palladium and palladium alloys emerge as promising substitutes for Ni in serving as a barrier or anti-corrosive layers. However, the considerable volatility in palladium prices underscores the imperative to explore and develop alternative solutions.

For these reasons, a new electroplating bath based on tin and ruthenium was formulated, which proved very valuable when palladium’s price exceeded 80 USD/g (compared to the 14 USD/g of ruthenium) [12]. This innovation has demonstrated significant value: apart from possessing anti-corrosive properties, Sn-Ru deposits exhibit resilience against solutions employed for stripping precious metals in defective deposits, thus preserving the entire underlying electroplating. This attribute proves advantageous as it saves time and resources for the electroplating industry, especially for decorative applications, and ensures the preservation of the whole electroplated structure. Notably, despite being an alkaline bath, it avoids hazardous compounds like cyanides. The previously addressed economic and environmental concerns have contributed to the increasing interest in pulsed current within recent years. Pulsed current has generated attention as a viable substitute for direct current due to its potential to provide various benefits related to deposit quality and properties [13,14,15,16]. For example, it has demonstrated a notable capacity to substantially reduce the amount of organic additives in acidic copper-plating baths [17]. In general, the flexibility of pulsed current allows for the selection of optimal waveforms and parameters to achieve the desired outcomes [18]. For instance, the pulsed reverse current technique, which introduces stripping time interrupting the plating current, has been shown to be effective in dissolving surface protrusions and obtaining very homogeneous surfaces [13,14]. This underscores the capability of pulsed current techniques to optimize electrochemical deposition processes and improve overall performance.

Regarding the electrodeposition of metal alloys, reverse pulsed currents can theoretically modify the composition of the alloy for the selective dissolution of one of the two metals during the anodic pulse. These aspects are particularly interesting to the jewelry and fashion accessories industry because, in addition to their other merits, pulsed and reverse pulsed methods can potentially facilitate the deposition of alloys featuring varied physical and aesthetic properties [19]. This capability enhances the industry’s flexibility in adhering to specific design requirements.

In this work, we investigate the application of pulsed deposition to a tin–ruthenium commercial bath. We performed some screening electrodepositions in direct, pulsed, and pulsed reverse currents. Electrochemical characterization measurements were carried out before sample preparation to understand the role of the main components. The formulation provides for a much higher tin content than ruthenium. Electrochemical measurements were therefore carried out first on the bath containing only tin or ruthenium and then on the complete bath. Tin is typically deposited from Sn(II) ions in an acidic bath and via Sn(IV) ions in an alkaline one, as in this case [20]. The electrodeposition from acid solutions consumes less electrical energy. It can offer faster deposition rates at ambient temperature. Still, it is more complicated to maintain, and the coating obtained is of poor quality, so organic additives are necessary to improve the efficacy of the deposit. Alkaline baths, on the other hand, can operate without additives but achieve a low maximum current density; moreover, they require high temperatures [21,22]. Industrially, ruthenium has long served as a hardening agent, especially for platinum or palladium, or as a catalyst in a few reactions [23,24,25,26]. However, there has been growing interest in its use as a substitute for other palladium group metals in recent years due to its relatively low cost [27]. Several studies have been conducted to optimize the electrodeposition of thin ruthenium layers to act as a barrier layer in the electronics industry [28,29] or of ruthenium alloys such as Ru-Co and Ru-Ni to be used as catalysts for different reactions [30,31,32,33]. Still, very few studies are related to the electrodeposition of Sn-Ru coatings [34] and, to our knowledge, none from alkaline matrices. Screening measurements were, therefore, conducted to understand the electrochemical functioning of the plating bath and the potential that the use of pulsed currents can offer. The waveforms used were selected based on literature data. The samples obtained were analyzed to evaluate the deposit’s thickness, composition, morphology, and color. The work focuses on applications in the decorative electroplating field, but the properties of the deposits obtained could also be studied for different industrial applications.

## 2. Materials and Methods

The Sn-Ru electroplating bath was prepared with the formulation provided by Valmet Plating srl (Calenzano, Italy): Sn 8 g/L (70 mM); Ru 250 mg/L (2 mM); SC (conducting salts) 30 g/L; ST (complex forming salts) 120 g/L; KOH 1 g/L. The two metals were supplied as, respectively, Na_2_Sn(OH)_6_ and ruthenium sulfamate, a complex commercial formulation that generally also contains Ru(III) as a trichloride [35,36]. Commercial electrodeposition of Ru usually occurs in electrolytes based on sulfamate because a good cathodic efficiency (>90%) and a crack-free coating can be obtained [37]. The compounds that can be used as conductive (SC) or complexing salts (ST) are listed in the US patent of the plating bath [12]. Still, the exact composition cannot be expressed here for reasons of industrial confidentiality. All the solutions were prepared using milli-Q water.

Electrochemical measurements were performed in a three-electrode cell: Ag/AgCl (KCl 3M) reference electrode (RE), gold rod counter electrode (CE), and a platinum or GC rotating disc electrode (RDE) with a diameter of 3 mm as a working electrode (WE). The experiments were performed at a temperature of 50 ± 1 °C in a double-walled cell by using a μAutolab3 (Metrohm, Herisau, Switzerland) and processed with NOVA 2.1 software. Before each new measurement, the platinum RDE was cleaned both mechanically and electrochemically following the procedure: polishing on diamond paste 0.5 μm and cyclic voltammetric (CV) sweeping from 1.4 V to −0.3 V in H_2_SO_4_ 0.5 M with a scan rate of 0.05 V/s.

As a substrate for the tin–ruthenium electrodeposition, brass plates measuring 5 × 3.5 cm^2^ (obtained by cutting Yamamoto Hull Cell plates into four parts) were used. The brass plates were previously electroplated in an industrial plant with a layer of Ni (10–12 μm) and then a thin film of Au (5–60 nm) to obtain smooth and easy-to-activate surfaces. The experiments were performed in a 0.5 L beaker using a mixed oxide electrode as an anode at 50 ± 1 °C, under stirring conditions (300 rpm). Additional samples were prepared in a Hull cell (Yamamoto) using the parallel sides of the cell to hold the substrate in a more fixed and reproducible position. For all the direct, pulsed, and pulsed reverse current electrodepositions, a Plating Electronic GmbH (Sexau, Germany) Pulse Reverse Power Supply was used. Before each electrodeposition, the surface of the samples was reactivated for 30 s using a commercial degreasing solution (30 s of degreasing, rinsing in demineralized water, rinsing in 5% w/w sulfuric acid, rinsing in demineralized water). The parameters for pulsed and reverse pulsed depositions were selected based on the literature of electrochemical measurements [14,19]. All of the pulsed current samples were prepared by imposing P = t_off_/t_on_ = 1. The duration of the electrodepositions was calculated to always pass 300 C/dm^2^ (value corresponding to 5 min of direct current deposition at 1 A/dm^2^). We tested four different t_on_ = t_off_ = 2, 5, 10, and 100 ms and two average current densities (j_AV_): 0.5 A/dm^2^ and 1 A/dm^2^. Therefore, the two j_ON_ were, respectively, 1 A/dm^2^ and 2 A/dm^2^ (j_ON_ = j_AV_ (P + 1)). Two DC samples were prepared at 1 and 0.5 A/dm^2^ to compare the results. As regards pulsed reverse current electrodepositions, the following was set: j_C_ (cathodic current density) = 3 A/dm^2^, j_A_ (anodic current density) = 0.5 A/dm^2^ and t_A_/t_C_ = 3, testing three t_C_ values: 2, 5, 10 ms (t_A_ = anodic pulse time; t_C_: cathodic pulse time). For pulsed reverse current depositions, j_AV_ was calculated with the following formula: j_AV_ = j_C_ × t_C_/(t_C_ + t_A_) − (j_A_ × t_A_)/(t_C_ + t_A_) [13], obtaining a value for all the samples of about 0.37 A/dm^2^. In all cases, the deposition time was 5 min.

The samples used for X-ray diffraction (XRD) analysis and X-ray fluorescence (XRF) maps were made with longer deposition times to achieve greater thicknesses—specifically, 10 min for direct and pulsed current samples and 20 min for pulsed reverse current samples. In order to keep the concentration of the two metals constant, periodic replenishments were made based on the thickness and composition of the deposits obtained (measured by XRF). Samples realized in pulsed current are indicated by the abbreviation PC followed by the pulse time (2, 5, 10 ms); samples realized in reverse pulsed current are indicated by the abbreviation PRC followed by the cathodic pulse time (2, 5, 10 ms); samples realized in direct current are indicated by the abbreviation DC.

The composition and thickness of the deposits were determined by both Energy Dispersive Spectroscopy (EDS) and XRF analysis. XRF measurements were performed with a Browman B Series XRF spectrometer (Schaumburg, IL, USA) using an acquisition time of 60 s, 50 kV tube voltage, 0.8 mA tube current, and a collimator of 0.6 mm in diameter. The thickness information was obtained with the FP method. For thicknesses of a few tens of nm, however, EDS analyses provide more accurate results. The measurements were performed with a Variable Pressure Hitachi SU3800 (Hitachi High-Tech, Tokyo, Japan), using an accelerating voltage of 10 kV and magnification of 300 for a total of 500,000 counts. The data were then analyzed with Aztec 2.1 software using the Layer Probe function, which uses the k-ratios method to measure thicknesses from 2 to 2000 nm.

Hitachi SU3800 was also used to acquire SE (Secondary Electron) and BSE (Backscattered Electron) images using an accelerating voltage of 10 and 5 kV.

The samples were also characterized by colorimetric measurements performed with a Konica Minolta (Tokyo, Japan) CM-700d portable spectrophotometer using the specular component included (SCI) mode, 10° observer, and D65 standard illuminant and averaging the value of three measurements acquired in the center of the sample.

Diffractometric measurements were conducted on three samples (DC, PC, PRC). In this case, the brass plates were previously electroplated in an industrial plant with a layer of Cu + Bronze (for a total of 10–15 μm) and then a thin film of Au (50–60 nm). The diffractograms were recorded with a Bruker New D8 Da Vinci (Bruker Italia srl, Milan, Italy) diffractometer (Cu-Kα radiation of wavelength = 1.54056 Å, 40 kV, 40 mA), equipped with an Euler cradle for massive samples and a Bruker LYNXEYE-XE detector (Bruker Italia srl, Milan, Italy). Scans were performed in a range of 2θ = 35–60°, with 2θ = 0.03° increment and 1 s of integration per step. To limit the substrate contribution, diffractograms were acquired with grazing angle geometry, setting θ: 2°. The ICDD (International Center for Diffraction Data) PDF-5+ 2024 database [38] was used for the attribution of the reflections.

## 3. Results

### 3.1. Electrochemical Analysis

**Figure 1:**
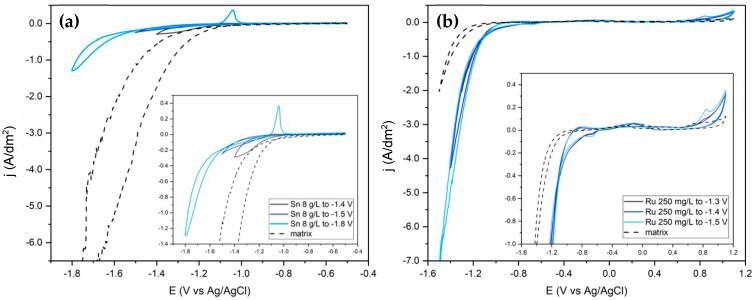
Cyclic voltammetries of the two metal components, 50 °C, 10 mV/s. Matrix: SC 30 g/L, ST 120 g/L, KOH 1 g/L; pH: 13.5. (**a**) Matrix with Sn 8 g/L, pH: 13.5. From −0.5 V to −1.4; −1.5; −1.8 V to −0.5 V. (**b**) Matrix with Ru 250 mg/L, pH: 9. From 0 V to −1.3; −1.4; −1.5 V to 1.1 V to 0 V. The Sn(IV) and Ru(III) oxidation peaks are at −1 V and +1 V, respectively.

Figure 1a shows the cyclic voltammograms of the bath containing only tin ions. The deposition of tin from alkaline baths containing stannate ions [Sn(OH)_6_]^2−^ occurs at very negative potentials. This makes the reduction peak difficult to detect due to the simultaneous hydrogen evolution reaction (HER). HER, however, acts as a levelling agent so that tin alkaline baths, unlike acid baths, generally do not require the addition of additives [39]. By extending the potential range down to −1.8 V, it is possible to observe the nucleation loop at about −1.6 V and the oxidation peak at −1 V. Figure 1b shows that the reduction of ruthenium occurs at more positive potentials and the nucleation (at about −1.2 V) is already observed by setting the minimum potential at −1.3 V. The oxidation peak at around 1 V, on the other hand, only appears from a minimum potential of −1.4 V. It should be noted that since the Ru sulphamate solution is acidic, the pH of the bath containing the matrix and the Ru sulphamate solution is lower than that of the matrix alone and that of the bath containing only Sn. This is also one of the reasons why the Ru content in the plating bath cannot be increased too much. The higher HER intensity is also due to the catalytic ability of ruthenium towards this reaction [37], which can also be observed using a GC such as WE (Figure S1, Supplementary Materials).

**Figure 2:**
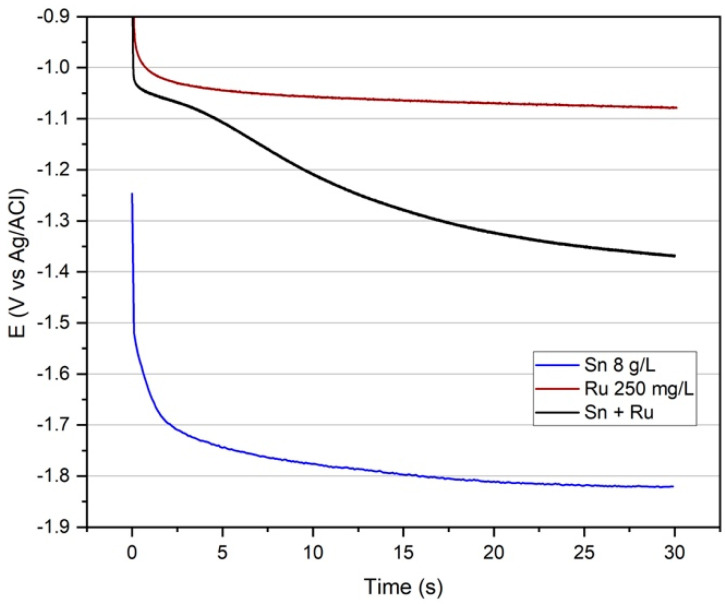
Chronopotentiometries of the electroplating bath containing all the components (black line) and all the components except Ru (blue line) or Sn (red line), J: 1 A/dm^2^, 50 °C, 30 s.

Chronopotentiometric measurements at 1 A/dm^2^ carried out in the complete bath and the two baths containing only one of the metals show a significant increase in the deposition potential due to the presence of Ru even if at a very low concentration (250 mg/L, Figure 2). It suggests the deposition of an alloy rather than the co-deposition of the two metals. However, the plot of the Sn-Ru system reaches the plateau potential in a longer time. It indicates the intervention of a phase change during deposition.

### 3.2. Characterization of Deposits

**Figure 3:**
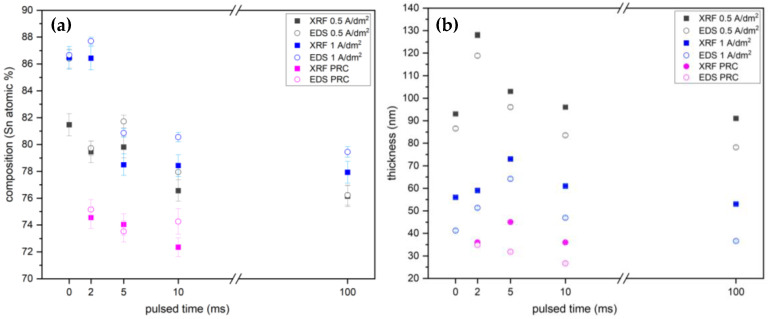
Composition (**a**) and thickness (**b**) of samples measured by both XRF and EDS techniques. DC samples were indicated with pulsed time = 0 ms; for PRC samples, the pulsed times correspond to the t_C_ times (2, 5, 10 ms). XRF data are expressed as the mean (and standard deviation) of three successive measurements made on the same point.

The Sn-Ru deposits were analyzed with EDS and XRF to determine their thicknesses and compositions (Figure 3). The greater thicknesses observed using an average current density of 0.5 A/dm^2^ can be attributed to a higher cathodic efficiency, which in turn is due to a lower hydrogen evolution. For both current densities, however, the PC samples with *t* pulses of 2 and 5 ms show greater thicknesses than the corresponding DC samples. At the same time, *t* pulses of 10 and 100 ms appear to be too long for the diffusive layer to pulsate and do not result in a significant change in the deposited thickness compared to the DC samples. It should be noted that the results obtained with the two techniques are very similar.

As expected, all PRC samples display lower thicknesses because of the lower average current density and, thus, the lower C/dm^2^ value. The PRC samples also exhibit the highest Ru content due to the preferential dissolution of tin during the anodic pulse. However, Figure 3 also suggests a correlation between composition and deposited thickness. This relationship has already been studied for Cu-Ag alloys by varying the DC deposition time. It can be attributed to the preferential deposition of the more noble metal in the initial deposition stages [40].

**Figure 4:**
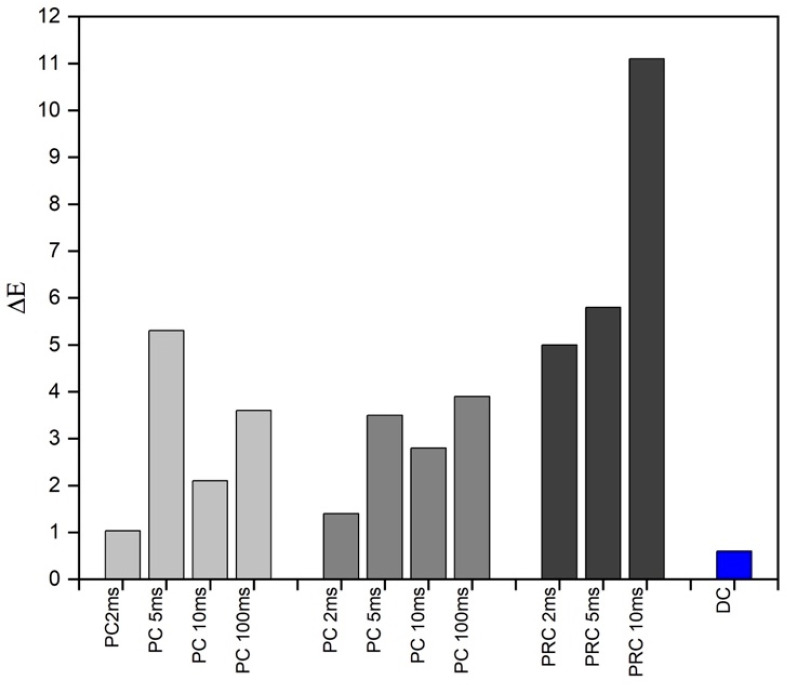
Colorimetric differences (ΔE $=\surd (L_{1}-L_{2}{)}^{2}+(a_{1}-a_{2}{)}^{2}+(b_{1}-b_{2}{)}^{2}$). The values were calculated and compared for the PC samples to the two corresponding DC samples (0.5 A/dm^2^: light grey and 1 A/dm^2^: grey). For the PRC samples (dark grey), the values were calculated with respect to the 1 A/dm^2^ DC sample. DC (blue) refers to the color difference between the two samples at 1 and 0.5 A/dm^2^.

**Figure 5:**
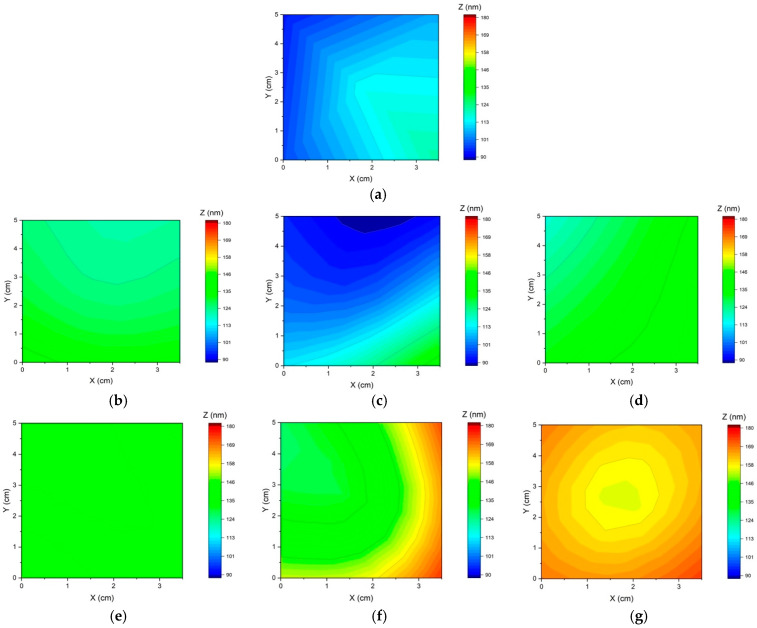
XRF thickness maps of the Sn-Ru deposit. (**a**) DC sample 1 A/dm^2^, (**b**) PC 2 ms, (**c**) PC 5 ms, (**d**) PC 10 ms, (**e**) PRC 2 ms, (**f**) PRC 5 ms, (**g**) PRC 10 ms. Data were acquired on the four corners (0.5 cm away from the edges) and in the center of the samples.

Because of the higher Ru content, the PRC samples also show a different color (observable to the naked eye, Figure S2, Supplementary Materials) due to the colorimetric CIE a* and b* coordinates taking on negative values. In contrast, the L values are almost constant. Color differences were calculated for the PC and PRC samples compared to the DC samples electrodeposited at the same (average) current density (Figure 4). In the CIE76 color space, the difference between two colors is indicated as ΔE and defined as the distance between two points in Euclidean space. According to the Schlapfer classification, 1 < ΔE < 3 indicates slight color differences, 3 < ΔE < 6 medium, and ΔE > 6 significant; in the electroplating sector, two colors are generally considered different for ΔE > 3 [41]. To assess the uniformity of the electrodeposited layer, XRF analyses were used to create thickness maps. The measurements were carried out on different samples realized on the same substrate but using a Hull cell (with the arrangement described in the Materials and Methods Section) to guarantee a more fixed position and lengthening deposition times (to deposit greater thicknesses, easily analyzable by XRF). Spectra were acquired at the four corners and in the center of each sample. As shown in Figure 5, the maximum variation in Z rarely exceeds 20–30 nm. However, the PRC sample with t_C_ = 2 ms shows a remarkably homogeneous thickness with a standard deviation of 2 nm over the five analyzed points (Table S1, Supplementary Materials). Moreover, PRC samples still show a lower Sn content despite the higher deposited thickness, indicating that the change in composition does not depend solely on the deposited thickness.

Figures S3 and S4 (Supplementary Materials) show the SE and BSE images acquired on the DC 1 A/dm^2^, PC with t = 2 ms and PRC with t_C_ = 2 ms samples together with the EDS-Maps to evaluate the morphology and the composition of the deposits. In all cases, nanostructured material of homogeneous composition was deposited, but the PRC sample showed a smoother and homogeneous surface with respect to the PC sample.

**Figure 6:**
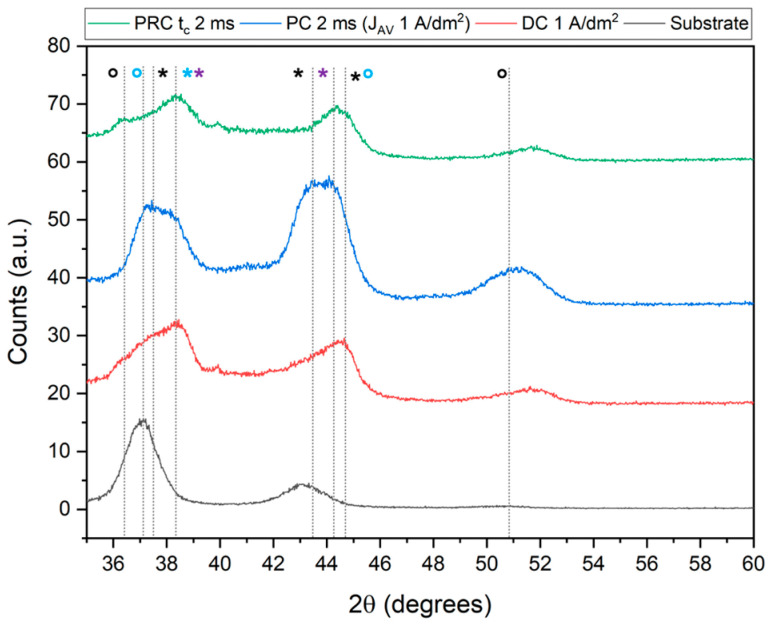
XRD patterns of Sn-Ru deposits obtained by using direct (DC), pulsed (PC with t = 2 ms), and pulsed reverse (PRC with t_C_ = 2 ms) current. Substrate: brass/Cu/bronze/Au; 2θ: 35–60°, increment: 0.03°, 1 s/step, grazing angle geometry. The attributions were made with the PDF-5+ 2024 database. Black: Sn (asterisk) and SnO_x_ (circle) reflections, light blue: Ru_3_Sn_7_ alloy (asterisk) and Ru_2_Sn_3_ alloy (circle), violet: Ru reflections (asterisk).

Finally, the diffractograms shown in Figure 6, acquired in grazing angle mode to limit the substrate contribution, reveal the presence in the deposits of both the single metals and Ru-Sn alloys: Ru_3_Sn_7_ (blue asterisk in Figure 6) and Ru_2_Sn_3_ (light blue circle in Figure 6).

The substrate shows the peaks of the underlying Au at 37.5° and 43.6° and bronze: Cu_3_Sn at 37.5° and 43.0°. However, an alloy with a higher Cu content can also be found: Cu_10_Sn_3_ due to the presence of some reflections at lower angles: 36.3°, 37.0°, and 37.4°.

Despite the width of the peaks, which can be attributed to both instrumental factors and the nanostructured nature of the deposit (Figure S3) comparing the diffractogram of the substrate with those of the samples, it is possible to attribute the reflections at 38.4° to the Ru_3_Sn_7_ alloy (light blue asterisk in Figure 6) and those at 37.2 and 44.6 to the Ru_2_Sn_3_ alloy (light blue circle in Figure 6) in addition to those of the single metals: Ru at 38.4° and 44.0° (purple asterisk in Figure 6) and Sn at 37.5°, 43.6°, and 44.6° (black asterisk in Figure 6). The reflections at 36.4° and 51.7° (black circle in Figure 6) can be attributed to, respectively, the cubic and tetragonal structures of SnO_2_.

## 4. Discussion

The electrochemical study of the alkaline tin–ruthenium electroplating bath is made particularly complex by the high pH values and the ability of ruthenium to catalyze the hydrogen evolution reaction. For these same reasons, the plating bath has a low deposition efficiency (~0.07 μm/min by using direct currents), which makes it unsuitable for the deposition of very thick layers. The electrochemical study of the plating bath made it possible to identify the deposition potential strongly influenced by the presence of ruthenium, even if in very small concentrations. Keeping P (t_off_/t_on_) = 1, it has been observed how pulse times of the order of a few ms (2 and 5 ms) can make the diffusive layer pulsate, allowing greater thicknesses to be deposited with the same C/dm^2^ and j_AV_. The use of reverse pulsed currents made it possible to obtain very homogeneous and uniform deposits, especially for t_C_ = 2 ms and a t_A_ = 6 ms. In all PRC samples, however, very different colors were obtained compared to the direct current deposits. This represents an important result for decorative applications because it suggests the possibility of using the deposit as a final layer. The composition does not vary much based on the waveform used for the deposition. Still, the Sn content decreases as the deposited thickness decreases and when PRC waveforms are used. Finally, from the diffractometric analysis, the deposition of the Ru_3_Sn_7_ and Ru_2_Sn_3_ alloys and the individual metals was observed for all the samples regardless of direct, pulsed, or pulsed reverse current use.

## 5. Conclusions

Pulsed current offers the possibility of obtaining different results depending on the waveform used, with great advantages in terms of economic and environmental sustainability for the electroplating industry. However, the wide variety of parameters on which it can act and the matrices to which it can be applied make the chemical and physical characteristics of deposits difficult to predict a priori. Preliminary studies are therefore necessary to identify the properties that can be modified for a specific matrix, testing different pulsed current waveforms. The study, in these terms, of a Sn-Ru bath provided very homogeneous deposits with different colors compared to DC deposits using reverse pulsed currents and greater thicknesses, with the same average current density, using pulsed currents with a pulse *t* of a few ms. The results suggest that the deposit can also be used as a final layer for decorative applications.

## Data Availability

The original contributions presented in the study are included in the article/Supplementary Materials, further inquiries can be directed to the corresponding authors.

## Supplementary Materials

The following supporting information can be downloaded at: https://www.mdpi.com/article/10.3390/ma17215326/s1.
