# Supplementary material for: Electrodeposition of Sn-Ru Alloys by Using Direct, Pulsed, and Pulsed Reverse Current for Decorative Applications
Source: Materials (Basel). 2024 Oct 31;17(21):5326. doi: 10.3390/ma17215326 (PMC11547184; doi:10.3390/ma17215326)
Supplement: Supplementary file 1 [file materials-17-05326-s001.zip › materials-3221256-supplementary.pdf]

Supplementary Information of

# Electrodeposition of a Sn-Ru alloys by using Direct, Pulsed and Pulsed Reverse current for decorative applications


**Margherita Verrucchi [1], Giulio Mazzoli [1], Andrea Comparini [2], Roberta Emanuele [2], Marco Bonechi [1,3], Ivan Del Pace [2], Walter Giurlani [1,3,*], Claudio Fontanesi [3,4], Remigiusz Kowalik[5], Massimo Innocenti [1,3,6,7*]**

1 Department of Chemistry "Ugo Schiff", University of Florence, Via della Lastruccia 3, 50019 Sesto Fiorentino (FI), Italy; e-mail@e-mail.com
2 Valmet Plating Srl, Via Erbosa 5, 50041 Calenzano (FI);
3 National Interuniversity Consortium of Materials Science and Technology (INSTM), Via G. Giusti 9, 50121 Firenze (FI), Italy;
4 Department of Engineering "Enzo Ferrari" (DIEF), University of Modena and Reggio Emilia, Via Vivarelli 10, 41125 Modena, Italy;
5 Faculty of Non-Ferrous Metals, AGH University of Krakow, al. Mickiewicza 30, 30-059 Krakow, Poland;
6 National Research Council-Organometallic Compounds Chemistry Institute (CNR-ICCOM), Via Madonna del Piano 10, 50019 Sesto Fiorentino (FI), Italy;
7 CSGI, Center for Colloid and Surface Science, Via della Lastruccia 3, 50019 Sesto Fiorentino (FI), Italy;
* Correspondence: W.G. walter.giurlani@unifi.it, M.I. m.innocenti@unifi.it


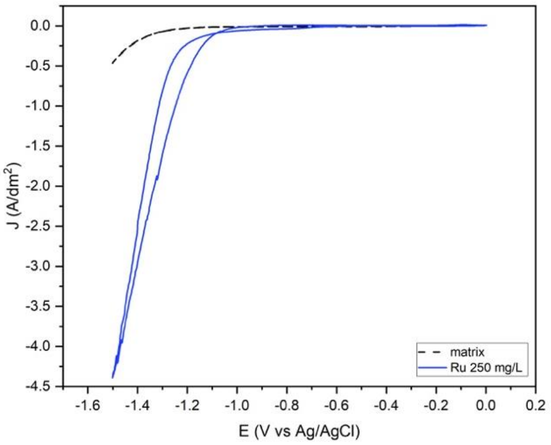


**Figure S1.** Cyclic Voltammetries of the galvanic bath containing all the components except Sn with a GC RDE as WE. From 0 V to -1,5 V to 0 V. Matrix: SC 30 g/L, ST 120 g/L, KOH 1 g/L, pH: 13.5. Ru 250 mg/L: matrix with Ru, pH 9, 50 °C, 10 mV/s.

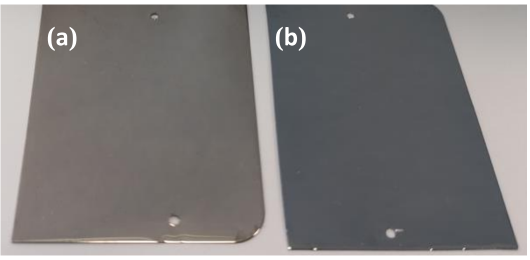


**Figure S2.** Color difference between the samples DC 1 A/dm² (a) and PRC 10 ms (b).

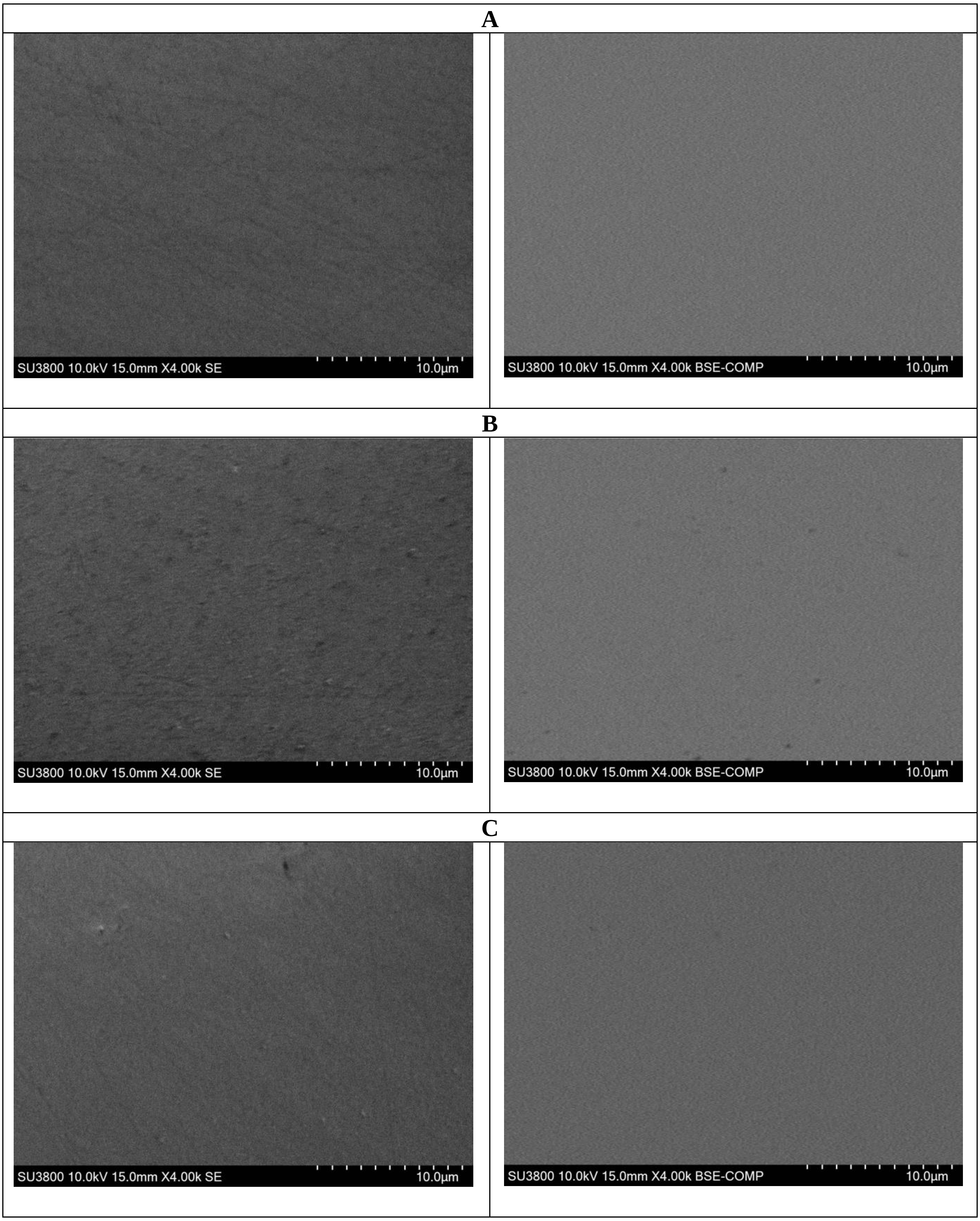


**Figure S3.** SE and BSE images. (A) DC 1 A/dm$^2$ (B) PC with t = 2 ms (C) PRC with $t_C$ = 2 ms.

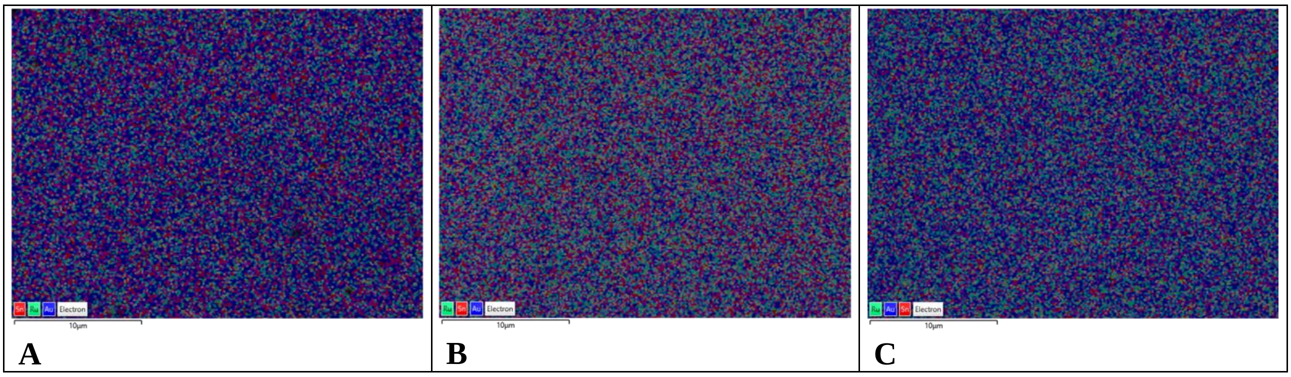


**Figure S4.** EDS Maps of samples in Figure S3 showing the elementary distribution of the two most superficial layers: Au(blue) and Sn-Ru (red-green). (A) DC 1 $A/dm^2$ (B) PC with t = 2 ms (C) PRC with $t_C$ = 2 ms. The Maps were acquired with an acceleration voltage of 5 kV to limit the contribution of the underlying layers.

**Table S1. Average value of thicknesses and compositions measured with XRF**

| Sample | Thickness (nm) | SD | Sn (At %) | SD |
|---|---|---|---|---|
| **DC** | 106 | 12 | 81.0 | 1 |
| **PC 2 ms** | 128 | 6.5 | 82.0 | 2 |
| **PC 5 ms** | 107 | 19.5 | 77.4 | 0.5 |
| **PC 10 ms** | 131 | 8 | 77.4 | 0.7 |
| **PRC 2 ms** | 142 | 2.5 | 76.0 | 2 |
| **PRC 5 ms** | 152 | 22 | 75.0 | 1 |
| **PRC 10 ms** | 166 | 7.5 | 74.5 | 0.8 |

**Table S1.** Average thicknesses and compositions measured on the samples shown in Figure 6. SD: standard deviations on the five measurements.
